# Senescence landscape in the liver following sepsis and senolytics as potential therapeutics

**DOI:** 10.1111/acel.14354

**Published:** 2024-10-23

**Authors:** Rupa Lavarti, Lun Cai, Tatiana Alvarez‐Diaz, Thalia Medina‐Rodriguez, Sergei Bombin, Raghavan Pillai Raju

**Affiliations:** ^1^ Department of Pharmacology and Toxicology, Medical College of Georgia Augusta University Augusta Georgia USA; ^2^ Georgia Cancer Center, Medical College of Georgia Augusta University Augusta Georgia USA

**Keywords:** aging, *Cdkn1a* ^*Cip1*^, dasatinib, quercetin, SASP, senescence, senolytics, sepsis

## Abstract

Senescence, caused by cell‐cycle arrest, is a hallmark of aging. Senescence has also been described in embryogenesis, wound healing, and acute injuries. Sepsis is characterized by a dysregulated host response to infection, leading to organ dysfunction and mortality. Most of the pathophysiology of human sepsis is recapitulated in the mouse model of polymicrobial sepsis, developed by cecal ligation and puncture (CLP). In this report, we demonstrate a rapid onset of cellular senescence in the liver of mice subjected to CLP‐induced sepsis, characterized by the upregulation of p21, p53, and other senescence markers, including SA‐βgal. Using RNAscope, confocal microscopy, and flow cytometry, we further confirm the emergence of p21‐expressing senescence phenotype in the liver 24 h after sepsis induction. Senescence was observed in several cell types in the liver, including hepatocytes, endothelial cells, and macrophages. We determined the landscape of senescence phenotype in murine sepsis by single‐cell sequencing, which further ascertained that this cell fate is not confined to any particular cell type but displays a heterogeneous distribution. Furthermore, we observed a significant reduction in mortality following sepsis when mice were treated with senolytics, a combination of dasatinib and quercetin, before the CLP surgery. Our experiments unequivocally demonstrated a rapid development of cellular senescence with sepsis and, for the first time, described the senescence landscape in the sepsis liver and the possible role of senescent cells in the worsening outcome following sepsis.

## Graphical abstract

This study demonstrates the onset of senescence in the liver following sepsis in a mouse model and describes the landscape of senescent cells in the sepsis liver. Treatment of mice with senolytics improved survival, suggesting that the senescence induction following sepsis is a critical factor in determining the outcome.

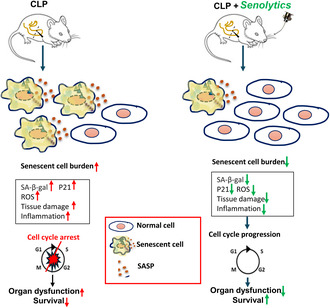

**Abbreviations**
ALT: alanine transaminase
AMDI: activation‐induced macrophage differentiation index
ANOVA: one‐way analysis of variance
CDK: cyclin‐dependent kinase
CLP: cecal ligation and puncture
CXCL‐1: chemokine (C‐X‐C motif) ligand‐1
D: dasatinib
DAPI: 4′,6‐diamidino‐2‐phenylindole
DDR: DNA damage response
EGF: epidermal growth factor
H&E: hematoxylin & eosin
ICU: intensive care unit
IFN: interferon
IL: interleukin
LPS: lipopolysaccharide
MCP1: monocyte chemoattractant protein‐1
MDA: malondialdehyde
MDMs: monocyte‐derived macrophages
MMP: matrix metalloproteinase
MPI: macrophage polarization index
MPO: myeloperoxidase
MSS: murine sepsis score
NO: nitric oxide
O/N: overnight
PBS: phosphate buffer saline
PI3K: phosphoinositide 3‐kinase
pRB: retinoblastoma protein
Q: quercetin
qRT‐PCR: quantitative reverse‐transcription polymerase chain reaction
RNA‐ISH: RNA in situ hybridization
ROS: reactive oxygen species
RT: room temperature
SAHF: senescence‐associated heterochromatin foci
SASP: senescence‐associated secretory phenotype
SA‐β‐gal: senescence‐associated beta‐galactosidase
SD: standard deviation
TNF: tumor necrosis factor
VEGF: vascular endothelial growth factor
WHO: World Health Organization

## 1 INTRODUCTION

Senescence, which results from permanent cell‐cycle arrest and is a hallmark of aging, was first defined by Hayflick and Moorhead in the early 1960s (Hayflick & Moorhead, 1961). Cellular senescence is observed not only with aging, but also in organismal development, maintaining tissue homeostasis, tissue remodeling and repair, acute injury, and in wound healing (Demaria et al., 2014; Muñoz‐Espín et al., 2013; Ramakrishna et al., 2012; Storer et al., 2013; Wiley & Campisi, 2021; Yun et al., 2015). For example, developmentally programmed senescence at birth was found to be essential to orchestrate postnatal lung remodeling (Yao et al., 2023). Senescence also has deleterious effects when it hinders tissue repair and regeneration, deplete stem/progenitor cell compartments, and contribute to tissue and organismal aging (Campisi et al., 2011; Coppé et al., 2010). While the pathogenesis associated with senescence is multifactorial, with autocrine and paracrine effects (Coppé et al., 2010), the role of the senescence process in the various pathologies is context‐dependent.

Sepsis is characterized by a dysregulated immune system leading to multiple organ dysfunction and death (Cai et al., 2023; Singer et al., 2016). The World Health Organization (WHO) considered sepsis as a global health priority and resolved to improve its prevention, diagnosis, and management (Reinhart et al., 2017). Despite major medical advances, sepsis remains the most common cause of death in intensive care units. Acute liver impairment often develops in the early stage of sepsis and significantly affects the severity and prognosis. Although studies have shown that multiple nonparenchymal cell lineages, such as neutrophils, endothelial cells, and Kupffer cells, interact in a complex way to cause acute liver dysfunction, it is still unclear how the cellular heterogeneity and dynamic regulation of these cell lineages contribute to the disease state (Dar et al., 2019). It is hypothesized that immuno suppression and immunosenescence might be associated with the progressive failure of organ function in sepsis (Martín et al., 2017) (Merdji et al., 2021; Monneret et al., 2021). The liver serves as a major metabolic organ responsible for preserving systemic homeostasis by regulating energy metabolism, xenobiotic and endogenous substance clearance, and of protein and amino acid metabolism. Hepatic senescence can significantly alter the microenvironment and tissue homeostasis, and has been shown to promote metabolic dysfunction of paranchymal and non‐paranchymal cells leading to increased vascular resistance, portal hypertension, steatotic liver disease, and cirrhosis (Sanfeliu‐Redondo et al., 2024). In this study, we used a murine model of polymicrobial sepsis to determine senescence induction and unravel its landscape in the liver (Cai et al., 2022, 2023). Acute liver impairment often develops in the early stage of sepsis and significantly affects the severity and prognosis (Strnad et al., 2017). Though previous studies have described a senescence phenotype with sepsis (Chen, Chen, et al., 2023; He et al., 2024; Lu & Lu, 2023), this is the first study demonstrating the heterogeneity of cell lineages in the liver undergoing sepsis‐induced senescence and the efficacy of senolytics in reducing mortality in a murine model of sepsis.

## 2 RESULTS

### 2.1 Sepsis promoted senescence phenotype

**FIGURE 1:**
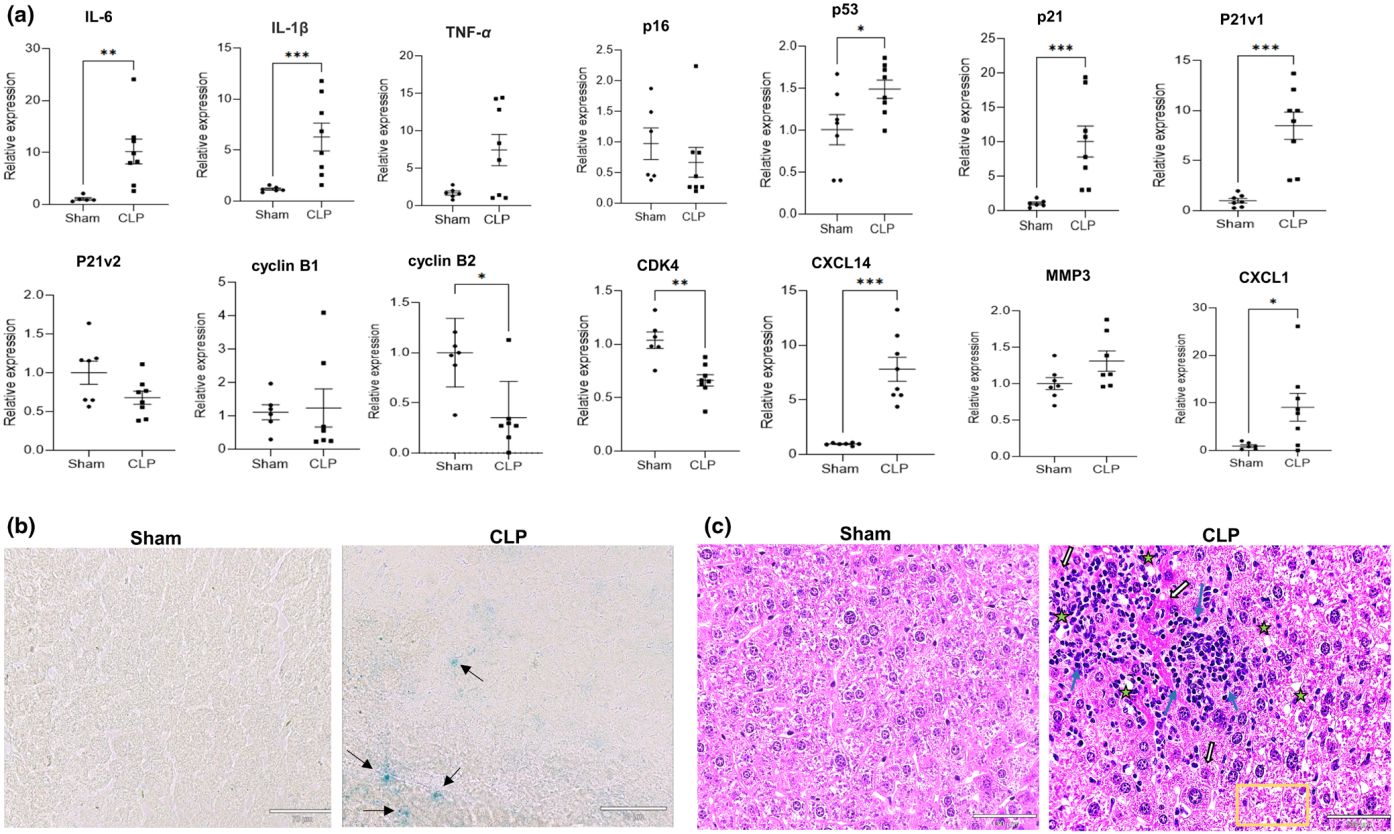
Cecal ligation and puncture (CLP) induces senescence in the mouse liver. (a) Assessment of SASP factors in sham and CLP liver by real time PCR at 24 h post‐surgery. An increase in inflammatory cytokines, senescent markers, SASP factors and down regulation of cyclin dependent kinases was seen in CLP liver. Gene expression was assessed by real‐time quantitative RT‐PCR relative to β‐actin and normalized to sham. Sham *n* = 7–8 animals; CLP *n* = 8 animals. Statistical analysis was performed using Mann–Whitney test with significance defined as *p < 0.05; **p < 0.01; ***p < 0.001. Data represents two technical replicates. (b) SA‐β‐gal staining. Liver cryosections sections (10 μm thickness) were fixed in fixation buffer for 10 min at RT. Washed with PBS for 2 min, three times followed by overnight incubation with X‐gal staining solution at 37°C. Intense SA‐β–gal signal (blue) was detected in CLP liver. Sham liver was negative for SA‐β‐gal staining. Stained slides were digitalized using an Echo Revolve microscope. Scanned slides were analyzed using ImageJ (NIH). For quantification of the staining in each group see Figure 6a (c) Histopathological evaluation of liver. H&E staining in sham liver represents a normal liver architecture. Liver damage with hemorrhagic necrosis (black & white arrow), microgranulomas consisting of infiltrated inflammatory cells (blue arrows), vacuolization (asterisk) ballooning and degeneration (yellow rectangle) was observed in CLP mice.

Our first experiment was to test whether sepsis induces a senescence phenotype. We chose to focus on the liver, as liver injury is observed within a few hours following sepsis induction and liver failure occurs early in sepsis (Li et al., 2018). We observed a significant increase in the gene expression of SASP markers, IL‐6, IL‐1β, TNF‐α, MMP3, CXCL1, and CXCL14 in the CLP liver, 24 h after the CLP surgery (Figure 1a). We also observed a significantly elevated expression of p53 and p21 (Cdkn1a; 10‐fold), and a reduction in the gene expression of CDK4, and cyclin B2 (Figure 1a). We found that increase in p21 expression during sepsis was predominantly contributed by the p21 variant 1 (p21v1), but not the variant 2 (p21v2) (Figure 1a). The systemic elevation of inflammatory cytokines was verified by multiplex cytometric bead array in plasma of CLP and sham mice. TNF‐α, one of the pro‐inflammatory cytokines, was significantly elevated in the plasma of mice subjected to CLP. CLP also resulted in the increased expression of IL‐1β, IL‐6, IL‐12, MCP1 and IL‐10 (Figure 6c). To further confirm the onset of senescence, we tested the classic marker of senescence, SA‐β‐gal, in the fresh frozen liver tissue from sepsis and control (sham) mice. Notably, intense SA‐β‐gal activity was found in the CLP liver, but not in the sham liver, demonstrating an enhanced senescence in response to sepsis (Figure 1b). Histological examination of the liver revealed tissue damage with hepatocyte swelling, loss of organization and structure, vacuolization, limited necrosis, and infiltration of immune cells in the liver (Figure 1c). SASP is also associated with the nuclear recruitment of phosphorylated γ‐H2AX, a typical marker of the DDR response, but was not observed in the CLP liver by IHC (Figure S1). Based upon the results we conclude that a senescent phenotype emerged rapidly following CLP‐induced sepsis in the mouse liver.

### 2.2 Increased p21 expression in the liver after CLP‐induced sepsis ‐ RNAscope

**FIGURE 2:**
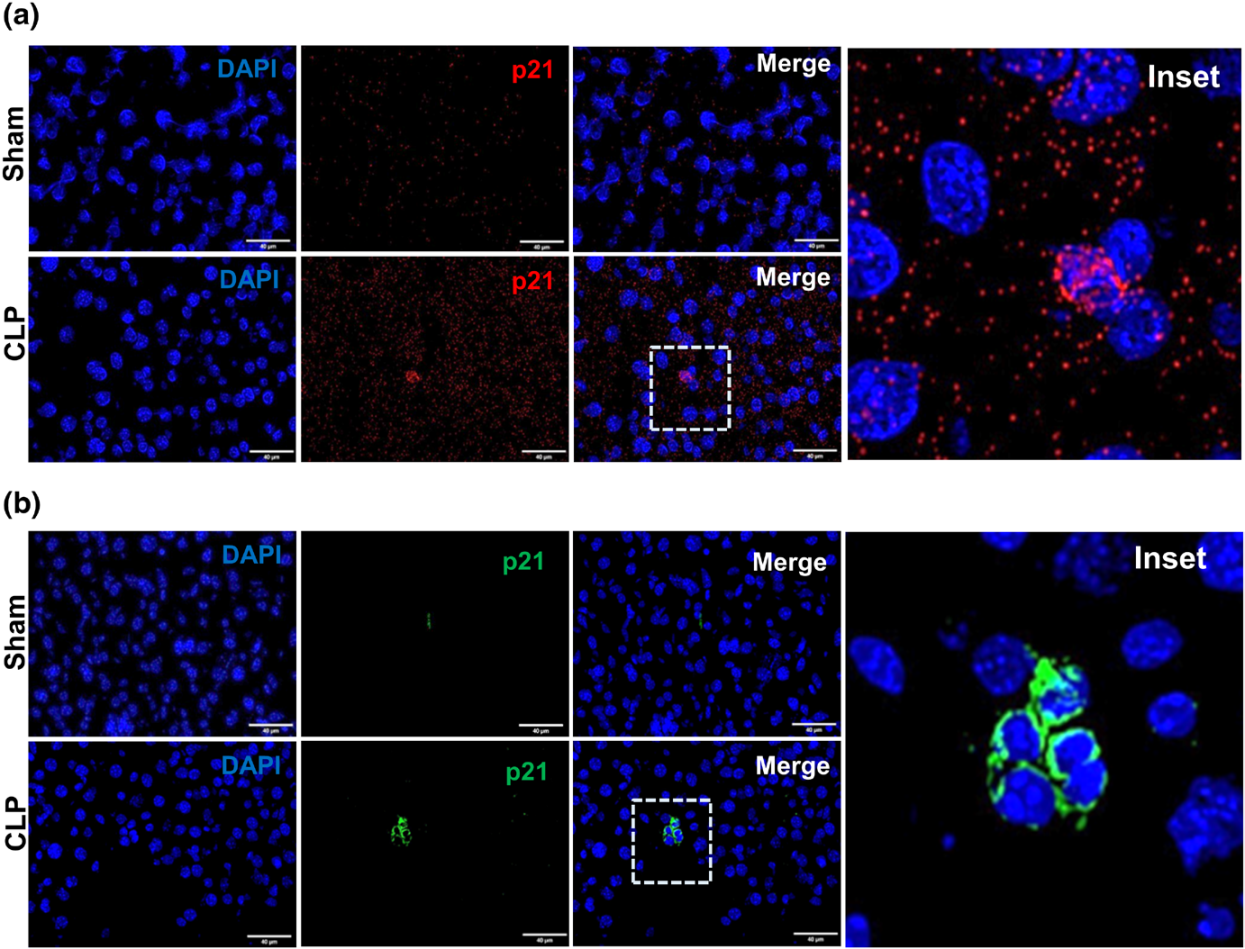
P21 expression in the mouse liver after CLP. (a) Validation of p21 expression by RNA in situ hybridization. Representative images showing p21 mRNA localization in sham and CLP liver. Nuclei counterstained with DAPI are seen in blue and hybridization with p21 probe is visualized as red punctate dots. Scale bar equals to 40 μm. (b) Immunofluorescence. p21 immunofluorescence in fresh frozen mouse liver from sham and CLP mice. Mice were euthanized and tissues harvested 24 h. following CLP procedure. p21 positive cells are in green with DAPI stained nuclei in blue. Dotted squares represent the inset.

To further establish p21 upregulation in sepsis liver and to evaluate the abundance of p21 mRNA levels in the CLP liver, we performed RNA‐in situ hybridization (RNA‐ISH), also called RNAscope (Figure 2a). In agreement with the initial findings using PCR, sepsis liver displayed profound expression of p21 mRNA transcripts with widespread dots and dot clusters indicating detectable p21 mRNA expression across the liver tissue at 24 h after sepsis induction. Using immunofluorescence we next tested and confirmed that elevated mRNA expression was consistent with molecular progression to protein expression of p21, with brightly stained clusters of cells expressing p21 (Figure 2b).

### 2.3 Senescence landscape in the liver after CLP‐induced sepsis

**FIGURE 3:**
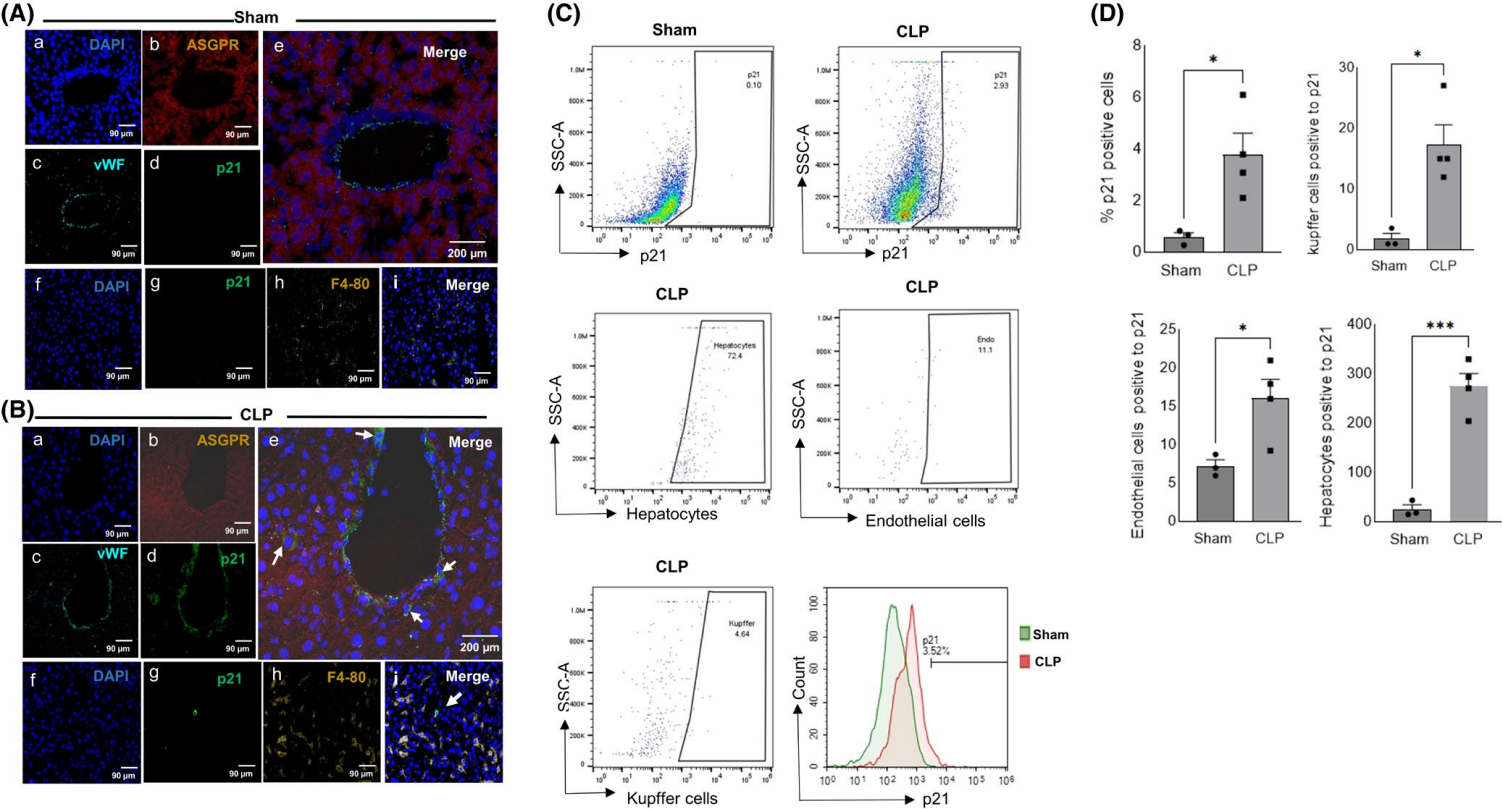
Sepsis induces senescence phenotype in the mouse liver. Representative images of triple immunofluorescence for p21, Von willebrand and asialoglycoprotein receptor (ASGPR) with DAPI in (A) Sham and (B) CLP liver. p21 (green) co‐localized with endothelial cells (cyan) and hepatocytes (red) (arrow) [a–e]. Double immunofluorescence for p21, and F4/80 with DAPI in CLP liver (f–i). p21 (green) co‐localized with Kupffer cells (brown) (arrow). Images out of three independent experiments. (C) Representative images of flow cytometry analysis. Single cell suspensions were prepared from liver by enzymatic digestion and using gentle MACS tissue dissociator. Debris was removed and single cells were stained with respective antibodies and gated based on SSC‐A/FSC‐A. Standard mean fluorescent intensity profiles show increased expression of p21 with sepsis. Sham liver single cells were used for negative gating of p21. Data were analyzed using FlowJo_v10.8.1. Images out of three independent experiments. (D) Quantification of p21 positive hepatocytes, endothelial cells and kupffer cells in the livers of sham and CLP mice. Statistical analysis was performed using unpaired *t‐*test with significance defined as *p < 0.05; ***p < 0.001.

**FIGURE 4:**
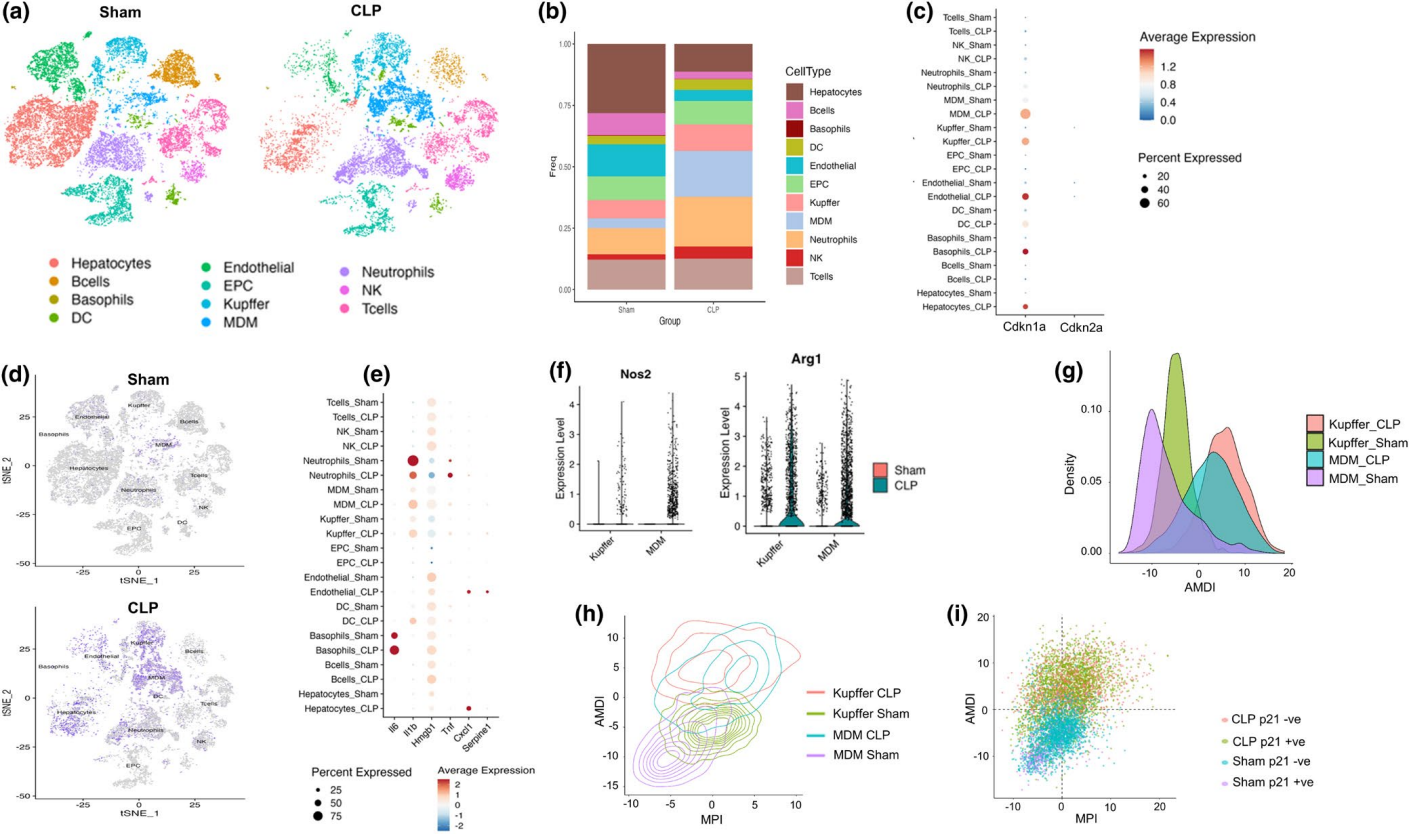
Transcriptomics landscape of sepsis liver. (a) The *t*‐distributed stochastic neighbor embedding (*t*‐SNE) plots indicate the separation of parenchymal and non‐parenchymal cells in sham and sepsis liver. (b) Bar chart showing the proportion of major liver subsets in sham vs. CLP. (c) Bubble map showing expression of p21 (Cdkn1a) and p16 (Cdkn2a) across 11 cell types. Dot color intensity represents the average expression level while size represents the percentage of cells within a cell cluster in which the marker was detected. (d) *t*‐SNE plots representing p21 (Cdkn1a) expression in sham vs. CLP liver. (e) Representative senescence signature gene expression of each cluster. (f) Violin plots of M1‐like and M2‐like macrophage marker expression in liver. MDM, monocyte derived macrophages; DCs, dendritic cells; EPC, Erythroid like and erythroid precursor cells; NK, natural killer cells. (g) Density plot of cell distributions along the AMDI scale. (h) Density contour plot displaying the relationship between MPI and AMDI scores. (i) Scatter plot of MPI and AMDI scores, differentiating between p21+ (positive) and p21‐ (negative) cells.

Given that CLP liver exhibited elevated p21 expression, our next objective was to identify the cell types expressing p21. Figure 3A,B (panels a–e) shows triple immunofluorescence staining against p21, endothelial cell marker von Willebrand factor (vWF), and hepatocyte marker asialoglycoprotein receptor (ASGPR) with colocalization of p21 in hepatocytes and endothelial cells. A double immunofluorescence staining of p21, and Kupffer cell marker F4/80 (Figure 3A,B (panels f–i)) revealed that p21 was not just confined to endothelial cells and hepatocytes, but a subset of p21 positive cells were also positive for F4/80. We subsequently verified these findings using flow cytometry, which revealed approximately 3% of the cells in CLP liver to be positive for p21 protein (Figure 3C). According to the FACS data, >70% of p21^+^ cells were hepatocytes, and endothelial cells and Kupffer cells constituted ~5‐10%. The above findings clearly indicate heterogeneity of senescent cells in the CLP liver. Single cell RNA sequencing (scRNA‐seq) allowed us to further determine and compare the differential expression of senescence marker genes p21 and p16 in various cell types as well as the sepsis‐driven change in non‐parenchymal cell population in the liver (Figure 4a–d and Figure S4). To account for the variability in sepsis response in individual mice, we pooled liver single cell preparations from 3 to 4 mice for each group. We were able to categorize the liver single cells into 11 major clusters as shown in Figure 4a,b. Sepsis resulted in a dynamic regulation of the ratio of non‐parenchymal cells in the liver, as seen by an increased influx of macrophages in the liver of mice (Figure 4a,b). Consequently, the relative proportion of hepatocytes, endothelial cells and B cells decreased with a slight increase in the NK cell population, with the pathological progression. p21 upregulation was predominantly observed in the liver macrophages, endothelial cells, basophils, and hepatocytes of CLP mice (Figure 4c,d), however, the expression of p16 was very minimal in all the cell types at 24 h following sepsis induction (Figure 4c and Figure S4). Around 60% of sepsis liver macrophages expressed p21 further demonstrating that (1) p21, but not p16 is the predominant senescence marker in sepsis, and (2) the senescence landscape is spread across different cell types in the liver (Figure 4a–e). Furthermore, we observed a shift in macrophage phenotype to M1 with sepsis induction (Figure 4F). The reduced expression of p16 compared to p21 was in line with our RT‐PCR gene expression results. When flow cytometry results were compared with RNA expression of p21, as profiled by scRNA‐seq, there were less protein expressing cells compared to mRNA expressing cells. This could be due to variability in RNA‐protein translation as well as the sensitivity of the two techniques. In addition, sepsis led to upregulation of SASP markers confirming the senescence signature following CLP (Figure 4e and Figure S6) and a net downregulation of differentially expressed genes in most of the cell types in the liver (Figure S7a,b).

We further analyzed the data to differentiate macrophages to be in polarized activation state or terminal maturation state, by a macrophage polarization index (MPI) and an activation‐induced macrophage differentiation index (AMDI) as described recently by Li and colleagues (Li et al., 2019). Figure 4g,h represents the macrophages in CLP and sham liver by AMDI, as well as MPI versus AMDI separating M1 (right) and M2 (left) macrophages. Further, when these cells were analyzed for the presence of senescence marker p21, most of the p21 expression was in newly emerged M1 or M2 population (Figure 4i).

### 2.4 D+Q attenuated sepsis severity, improved survival, and reduced SASP burden

**FIGURE 5:**
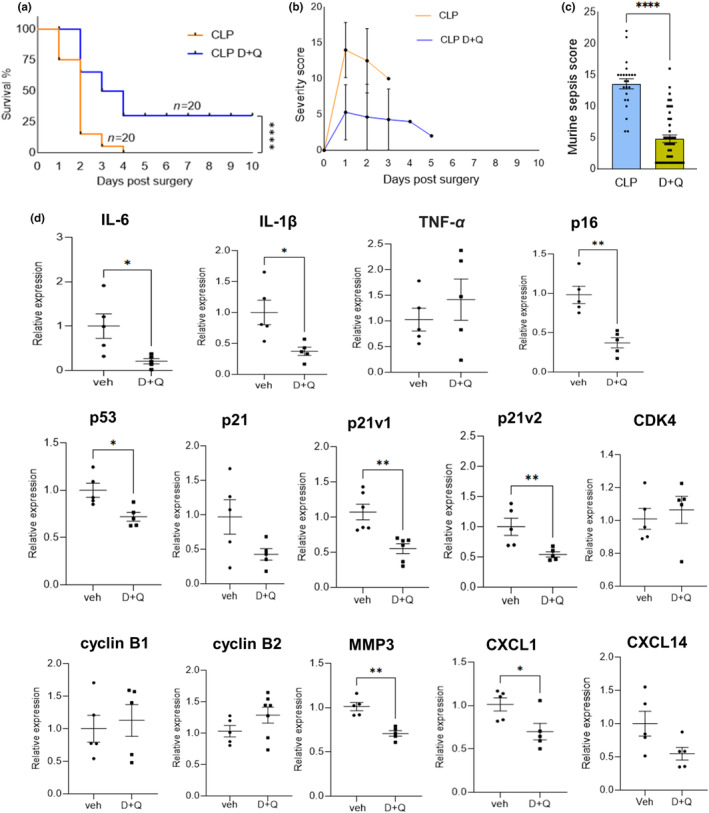
Effect of senolytics on survival and senescence markers in CLP‐induced sepsis. (a) Kaplan–Meier survival plot of CLP and D+Q administered mice, a 10 day survival study (*n* = 20 mice per group). An increase in the survival time for D+Q administered mice (5 mg/kg dasatinib and 50 mg/kg quercetin) compared to mice without D+Q administration was observed. *p* < 0.0001—As per log‐rank Mantel‐cox test and *p* = 0.0001 as per Gehan‐Breslow‐Wilcoxon test. (b) Murine sepsis score (MSS) of mice over time. A two tailed non‐parametric Mann–Whitney test was used in C depicting a significance of *p* < 0.0001. (c) Day1 sepsis score. *n* = 18 for CLP and *n* = 20 for CLP D+Q. (d) Assessment of the expression of SASP factors in livers of CLP mice administered with vehicle (DMSO) in comparison with CLP mice administered with D+Q. CLP vehicle *n* = 5; CLP D+Q *n* = 5. CLP mice administered with senolytics had decreased levels of inflammatory cytokines, senescent markers, SASP factors and upregulated cyclin dependent kinases. Gene expression was assessed by real‐time quantitative RT‐PCR relative to β‐actin and normalized to vehicle. Statistical analysis was performed using Mann–Whitney test and data are represented as mean ± SEM. *p < 0.05 and **p < 0.01 and ****p < 0.0001 represents significance.

Our experiments demonstrated a wide landscape of senescence induction following sepsis. To determine whether the newly emerged senescent cells contribute to the clinical outcome or pathology, we treated mice with D+Q, prior to sham or sepsis surgery. We observed a high mortality rate in animals that underwent CLP with no survival by Day 4 (Figure 5a). Remarkably, the survival rate was significantly higher in D+Q treated mice, with a 30% survival in 10 days compared to 100% mortality by 4th day after the CLP surgery. Furthermore, vehicle‐treated CLP mice displayed a higher sepsis score at 24 h. post‐surgery compared to the mice treated with D+Q (Figure 5b,c).

**FIGURE 6:**
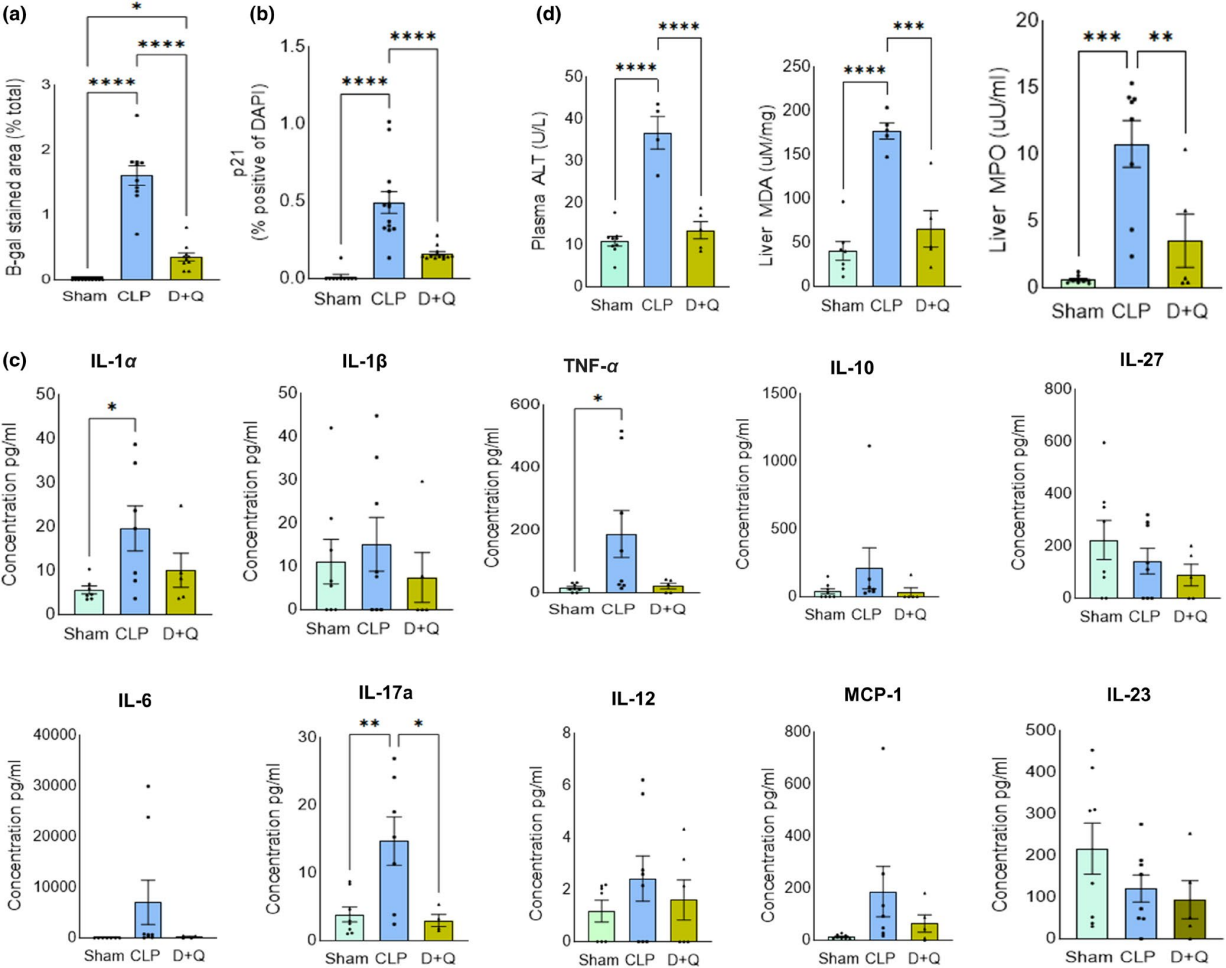
Effect of senolytics on liver injury and inflammation in CLP induced sepsis. (a) Quantification of SA‐β‐gal. Reduced SA‐β–gal intensity (blue) was detected in D+Q administered CLP liver. Data shown are mean ± SEM of three experimental repeats. (b) Quantification of p21 positive cells. Data are presented as means ± SEM. Unpaired two‐tailed *t‐*test, **p < 0.01, ***p<0.001, ****p < 0.0001 (c) Multiplex analysis of cytokines and chemokines in blood plasma. Cytokines in Sham, CLP and CLP D+Q mice were measured using multiplex cytometric bead array in the blood plasma at 24 h following CLP. *n* = 5–8 mice per group. Plasma concentrations of inflammatory cytokines were elevated in CLP mice while mice administered with D+Q attenuated this effect. IL, interleukin; TNF, tumor necrosis factor; MCP1, Monocyte Chemoattractant Protein‐1. Ordinary one‐way ANOVA was used for analysis. Results are given as mean ± SEM. *p < 0.05. (d) Effect of senolytic drugs on sepsis induced liver injury. Levels of ALT in plasma, MDA and MPO activity in liver 24 h after surgery. ALT, MDA and MPO depressed significantly in D+Q administered mice indicating protective nature of D+Q. Sham (*n* = 7), CLP (*n* = 5) and CLP mice administered with D+Q (*n* = 5). Statistical analysis: One‐way ANOVA with results given as mean ± SEM. ****p < 0.0001.

We also examined the impact of senolytic treatment on the expression of SASP factors by assessing gene expression using RT‐PCR. Compared to CLP mice, we noticed a marked reduction in the expression profiles of various SASP factors in the liver, IL‐6 and IL‐1β, were significantly reduced with D+Q treatment (Figure 5d). The classical biomarkers of senescence, p21 and p16, exhibited a significant down regulation. Interestingly, both variant 1 and variant 2 of p21 were decreased with D+Q treatment (Figure 5d). Though not significant, the expression of cyclin dependent kinase CDK4, and cyclins B1 and B2 were improved (Figure 5d). We next assessed the therapeutic potential of D+Q by evaluating the pathological damage of the liver by H&E staining. D+Q reduced liver injury induced by CLP sepsis, indicating protection from sepsis‐associated organ damage (Figure S3a). The senolytics treatment decreased senescence burden as seen by significant reduction in SA‐β‐Gal (Figure 6a and Figure S3b). RNA‐FISH confirmed a low to moderate expression of p21 mRNA transcripts in D+Q treated mice (Figure S3C) and a reduction in p21^+^ cells was observed with IHC (Figure 6b and Figure S3D). D+Q treatment also reduced systemic levels of most cytokines tested (Figure 6c). In addition, D+Q treatment significantly lowered the plasma ALT levels, a hallmark of liver injury in CLP mice (Figure 6d). MPO and MDA activity were also decreased indicating reduced oxidative stress following the senolytic treatment (Figure 6d).

To further test the therapeutic efficacy of D+Q with aging, we evaluated sepsis‐associated liver injury in aged mice (24 months old) subjected to CLP surgery (Figure S2). As expected serum ALT, and liver MPO, MDA were increased with CLP. Though treatment with senolytics, D+Q, reduced the levels of ALT, MDA, and MPO in aged mice, a significant reduction was observed only in MDA levels. We conclude that in aged animals, the dose and frequency of D+Q administration might have to be increased to observe a more significant functional difference. Altogether, these findings indicate the potential of D+Q in reducing the pathology associated with sepsis as well as markers of senescence in the liver.

## 3 DISCUSSION

While senescence is well‐described as a hallmark of aging (Campisi, 2013; López‐Otín et al., 2013) and contributes to aging pathology, the function of senescent cells is context specific or unknown in many other conditions (Baker et al., 2011; Campisi, 2005; Smith‐Vikos & Slack, 2012; van Deursen, 2014). Unlike in chronic diseases, a rapid emergence of senescent cells was reported in several other conditions (Blazquez‐Prieto et al., 2021; Chu et al., 2020; Lopez‐Dominguez et al., 2021; Yao et al., 2023). In the present study, we sought to establish the acute induction of senescence in a murine model of sepsis, determine the senescence landscape in a critical organ, the liver, and to examine whether removal or reduction of these cells alter the trajectory of sepsis.

There is no single, universal, or model‐specific biomarker to identify senescent cells (Campisi, 2013; Sharpless & Sherr, 2015) making the study of in vivo senescence challenging (McHugh & Gil, 2018). However, the key cyclin‐dependent kinase inhibitors, p16 and p21, are upregulated in senescent cells and often serve as markers of senescence. It is hypothesized that p21 is activated early on senescence entry and maintains the senescence phenotype, while p16 is a late expression marker (Dulić et al., 2000). A similar effect of elevated p21 expression, but not p16, with rapid onset of senescence following hemorrhagic shock was recently reported (Chu et al., 2020). Similarly, an increase in p21expression was observed in mouse bones within 24 h following radiation injury, whereas p16 levels increased gradually over the course of several weeks (Chandra et al., 2022). Some reports attribute p16 in replicative senescence (Mirzayans et al., 2012) and p21 in stress‐induced senescence (Luo et al., 2011). Using a combination of molecular techniques, we demonstrated the induction of senescent cells in response to sepsis, unraveled the senescence landscape in the liver, and observed a significant reduction in mortality after sepsis when treated with the senolytics, D+Q.

### 3.1 CLP induced liver injury and elevated SASP profile

The increase in liver enzyme ALT and oxidative stress markers MDA and MPO confirmed sepsis‐induced liver injury. The liver from sepsis mice displayed upregulated gene expression of p21 (about 10‐fold), with a decline in the expression of cyclins and cyclin dependent kinases. We also detected elevated levels of p21 variant 1 (p21v1), but not variant 2 (p21v2), indicating that sepsis‐induced senescence is regulated by the p21v1, not v2. This finding is in line with a study recently published by Campisi group demonstrating an upregulation of p21 v1 transcript in response to genotoxic stress while p21 v2 increase to be associated with aging (Lopez‐Dominguez et al., 2021). SASP profile at 24 h. of sepsis induction revealed an exacerbated inflammatory response evidenced by increased tissue levels of proinflammatory cytokines IL‐6, IL‐1β and TNF‐*α*. The inflammatory factor MCP‐1, which regulates the infiltration of immune cells to the inflammatory lesions was elevated several fold in the blood in sepsis mice (Wang et al., 2008). Sepsis also elevated MMPs, which react to tissue injury and drive sepsis associated inflammation (Jones et al., 2022; Zinter et al., 2019). Further, we noticed a 10‐fold increase in the levels of chemokines CXCL1 and CXCL14 in the CLP liver. Consistent with the elevated SASP levels in the liver, a systemic proinflammatory response was also observed in blood plasma.

To characterize the landscape of senescence cells in sepsis liver, we used p21 as the lead marker and first tested its localization in the liver by RNAscope which revealed an increased and widely scattered expression across the liver tissue. A more localized expression pattern was observed for the p21 protein as observed by immunohistochemistry. To further quantify p21 positive cells in the sepsis liver, we performed flow cytometry of single cell preparation of the liver. At 24 h. after CLP, hepatocytes formed most of the p21^+^ cells, followed by endothelial cells, and Kupffer cells.

Inflammation and senescence are integral parts of aging and age‐associated co‐morbidities and are interconnected processes that can exacerbate one another, leading to a decline in tissue function and organismal fitness (Campisi et al., 2019; Fulop et al., 2017; Sayed et al., 2021). Even though one of the characteristics of senescence is SASP, in vitro and in vivo studies have shown that inflammation can trigger senescence (Lasry & Ben‐Neriah, 2015; Li et al., 2023; Vernot, 2020). A profound inflammatory response is observed following CLP and this exacerbated systemic immune hyperactivation can be a causative factor in senescence induction (Li et al., 2023; Wiersinga & van der Poll, 2022). Though most organs are affected by this process, our focus in this study was the liver, as liver dysfunction is well described in sepsis (Beyer et al., 2022; Woznica et al., 2018). Senescent cells lose their capacity to multiply or regenerate, impairing the ability of the liver to repair itself after sepsis‐induced insult (Huda et al., 2019). In addition, the SASPs secreted by the senescence cells can impart paracrine effects in the liver. Furthermore, mitochondrial dysfunction is a hallmark of sepsis and dysregulation of mitochondrial dynamics (fusion and fission processes), function and metabolism is observed in senescent cells (Abate et al., 2020; Cai et al., 2022; Martini & Passos, 2023). The mitochondrial functional impairment affects a number of vital processes in the cell, including ATP‐requiring metabolic functions, further intensifying liver dysfunction and organ damage. The observed senescence in Kupffer cells and monocyte‐derived macrophages (MDMs) in the liver can also be detrimental to liver function, as senescent macrophages have been shown to produce excessive pro‐inflammatory cytokines, reduced phagocytic ability and impaired antigen presentation (Franceschi et al., 2000). This may also adversely affect their function in resolving inflammation. Therefore, the CLP‐induced inflammation can trigger the senescence phenotype, and the induction of senescence accentuates functional impairment of the liver.

### 3.2 Transcriptomics landscape of senescence in CLP liver

The utilization of scRNA‐seq allowed us to better understand the heterogeneity and complexity of the senescence landscape. Several studies involving chronic liver diseases from mice and humans have been reported at single cell level (Chen, Ren, et al., 2023; Ramachandran et al., 2019; Terkelsen et al., 2020; Zheng et al., 2017). However, none in the context of senescence signature in sepsis. The comprehensive transcriptomic analysis of CLP liver using scRNA‐seq confirmed the upregulation of p21 with minimal increase in p16, as evidenced by real‐time PCR. The largest population of cells with p21 expression was found in the liver macrophages, followed by hepatocytes, endothelial cells, basophils, and neutrophils demonstrating a rapid development of cellular senescence in most of the cell lineages in the liver. We also observed a marked change in the ratio of different cell lineages in the liver with macrophages representing a significant proportion of cells after sepsis. The CLP treatment has clearly induced macrophage reprogramming as we see differential levels of M1 and M2 markers following sepsis. Nos2 positive MDMs were almost non‐existent in the sham mice, while a large population of this subtype appeared within 24 h of CLP. Whereas, there was a certain noticeable fraction of pre‐existing Arg1‐positive M2 macrophages in the sham, this subpopulation also amplified following CLP surgery, but not to the extent of M1 fraction.

Recently, Li, Zhou, and colleagues developed algorithms that can differentially identify macrophage subpopulations by polarized activation state and terminal maturation state (Li et al., 2019). Using these algorithms, they deduced a MPI and an AMDI to differentially identify each cell to be in an inflammatory or in a terminal maturation state (Li et al., 2019). We analyzed our data using their algorithmic framework and observed a clear separation of macrophages in sham and CLP liver by AMDI (Figure 5h). Li et al. used an MPI versus AMDI plot (which they called a MacSpectrum) wherein the M2 like macrophages remain to the left of the plot and M1‐like to the right. In our results, while the sham macrophages mostly remained in the lower left quadrant, the CLP macrophages moved to the top right, indicating a high level of polarization to the M1‐line. The Kupffer cells also demonstrated a similarly high AMDI and MPI with sepsis. The M1‐like transformed cells, according to Li et al. (2019), move to the right quadrants, with differentiated M1‐like macrophages occupying the top right corner. The lower MPI suggests less inflammatory or more M2‐like states. In acute infection following exposure to the cecal exudate, monocytes are recruited to the liver, and together with the resident macrophages (Kupffer cells), undergo terminal differentiation and activation. This suggests that the onset of senescence in the liver can be a driving factor for the tissue influx of macrophages. In a recent study, it was shown that p21, through Rb hypophosphorylation, induces CXCL14, a macrophage attractant chemokine, thereby recruiting and differentiating macrophages to the senescent tissue foci (Sturmlechner et al., 2021). CXCL14 was abundantly expressed in the liver following CLP‐induced sepsis (Figure 1). This data suggests that the tissue inflammation and senescent induction form a vicious cycle in sepsis, with one exacerbating and reinforcing the other.

These data therefore demonstrate CLP‐induced macrophage reprogramming resulting in a dominant M1 phenotype by 24 h following the induction of sepsis. Furthermore, as seen in the t‐SNE plot, after CLP there was a clear increase in the levels of senescence marker p21 in the macrophages, in both MDM and Kupfer cells, indicating that macrophage senescence can play a role in the CLP‐induced inflammation. Though senescence did not spare any subpopulation of the cells in the liver it is unclear whether senescence of any specific cell type is more deleterious than the other cell types in the liver and needs further investigation. These results show that while tissue inflammation following sepsis can trigger a senescent phenotype, the converse is also possible as senescent cells secrete SASP including CXCL4 and trigger an influx of inflammatory cells. Whether senescent cells are the cause of tissue inflammation or the inflammation together with impaired microcirculation cause senescence in sepsis, ablation of senescence cells will demonstrate the functional role of these cells in outcome following sepsis.

### 3.3 Senolytics improved survival and suppressed senescence burden

Senolytics have been shown to remove or inactivate senescent cells and alleviate a variety of senescence‐related phenotypes including local and systemic SASP activation. In pursuing this idea, we assessed the efficacy of senolytic drug combination D+Q in reducing the symptoms and mortality associated with sepsis. We observed lower sepsis scores and reduced mortality in mice treated with D+Q. We noticed a decrease in SASP and a trend toward higher CDK levels with D+Q treatment. Importantly, transcriptional levels of p21 and p16 in D+Q treated liver were significantly down regulated compared to that in vehicle treated mice. D+Q diminished the secretion of CLP‐induced SASP. Clearance of senescent cells by D+Q was further demonstrated by the detection of low levels of p21 mRNA transcripts using RNA‐FISH. Additionally, we investigated whether the relevant gene expression followed a similar trend by proteins as detected by IHC. Compared to vehicle‐treated CLP mice, D+Q dramatically decreased the relative number of p21 protein expressing senescent cells. A recent study demonstrated increased survival and reversal of age‐associated physical dysfunction with intermittent oral administration of the senolytic cocktail D+Q (Xu et al., 2018). This is also in line with the studies which demonstrated a decrease in SASP factors with the genetic clearance of p16 expressing senescent cells in aged p16‐INK‐ATTAC mice (Farr et al., 2017) as well as with the pharmacological clearance of senescent cells by D+Q in radiation‐related bone loss (Chandra et al., 2020). In another study an increase in p21expression in mouse bones was observed within 24 h following radiation whereas p16 expression increased gradually over the course of several weeks (Chandra et al., 2022). Similarly, in cultured mouse dermal fibroblasts, levels of p21 mRNA were induced within 3 h of radiation, whereas p16 transcript levels elevated much later, ~50‐h post‐radiation (Lopez‐Dominguez et al., 2021). Thus, the early induction of p21 following sepsis would be consistent with the hypothesis that p21 pathway is the primary driver of cellular senescence in stress‐induced senescence.

### 3.4 D+Q improved liver function

The livers of D+Q treated mice presented a normal histology. D+Q significantly reduced the oxidative stress caused by sepsis and improved liver function as evidenced by the diminished levels of liver ALT, MDA, and MPO. Similar effect was reported in a murine model of non‐alcoholic fatty liver disease, with dasatinib effectively reducing liver steatosis, inflammation, fibrosis, and hepatocellular ballooning, by attenuating lipogenesis, and inducing M2 macrophage polarization with antifibrotic activity (Elsayed et al., 2021). Another significant finding from our study is that livers derived from D+Q treated mice showed a decrease in SA‐β‐gal staining though it was not completely abrogated. These findings are consistent with two other separate studies that showed D+Q was effective in lowering the expression of p16 and SA‐β‐gal in patients with diabetic kidney disease and idiopathic pulmonary disease during a phase I clinical trial (Hickson et al., 2019; Justice et al., 2019). This drug combination demonstrated senolytic effects in white adipose tissue, with a decrease in the expression of SA‐β‐gal, p16, and p21 (Murakami et al., 2022). Though we treated the mice with D+Q one day prior to sepsis induction, the effect of senolytics treatment likely persisted at least for 48 h. It is also likely that the early intervention was the cause of reduced mortality observed in D+Q treated groups which lead us to speculate that senescence may be a contributing factor in the increased mortality observed after sepsis in the elderly, whose senescence burden is high. To test the effect of senolytics in the aged mice, we treated aged mice with D+Q before performing CLP surgery using a protocol similar to that was done for the young mice. Though plasma ALT, MPO and liver MDA levels were reduced with the D+Q treatment, a significant reduction was observed only in MDA levels. We conclude that in aged mice, the dose and/or frequency might have to be increased to observe a significant functional difference, due to confounding factors such as age‐associated senescence.

Our results show induction of senescence in several cell types in the liver, including hepatocytes, endothelial cells and neutrophils, while hepatocytes form the liver parenchyma, endothelial cells are critical in maintaining vascular tone and regulating blood flow. The onset of senescence in endothelial cells not only results in the functional impairment of the vessels, the senescent endothelial cell‐derived SASPs can cause more vascular damage. Similarly, neutrophil senescence may lead to impaired pathogen clearance and increased organ damage. The senescent neutrophils may not be cleared easily from the liver and they contribute to further liver dysfunction and damage. Studies have also showed that the senescence process in acute liver injury may be therapeutically possible by SASP inhibition (Bird et al., 2018). It may therefore be inferred that senolytics could have beneficial effects in the context of sepsis and our observation of improved liver function and animal survival when treated with D + Q is consistent with this explanation.

In conclusion, our study shows that sepsis results in the development of a senescence phenotype in young mice. Importantly, scRNA‐seq not only confirmed the upregulation of p21in sepsis liver, but also revealed the sepsis‐induced senescent landscape in the liver for the first time. Sepsis altered the ratio of non‐parenchymal cells in the liver with a large infiltration of macrophages. Senolytic treatment with D+Q significantly improved survival following sepsis and reduced senescence molecular profile while also improving liver morphology. Altogether, our results indicate that CLP‐induced sepsis drives senescence in a p21 dependent manner and eliminating these cells through senolytics treatment prevents downstream consequences of sepsis. Our findings advanced the fundamental knowledge in host‐response to sepsis, opened a new strategy for the treatment of ensuing organ dysfunction, and established senescence as a target in the treatment of sepsis.

## 4 METHODS

### 4.1 Animals

Animal experiments in the study were approved by the Institutional Animal Care and Use Committee (IACUC) at Augusta University (AU). C57BL/6 male mice of ages 10–12 weeks were obtained from Charles River Laboratories International, Inc. (Wilmington, MA). Animals were housed in AU animal facility during the experiments as per the relevant guidelines and regulations.

### 4.2 Sepsis induction

CLP surgery was performed as described earlier by Cai et al. (2022). Briefly, mice were anesthetized with 2% isoflurane and analgesia was provided by a subcutaneous injection of carprofen 2 mg/kg of body weight (Covetrus, UK). After a 1 cm midline laparotomy, cecum was exposed, ligated, and perforated with a 22‐gauge needle, gently squeezing a small amount of feces. The cecum was returned into the peritoneal cavity and the incision closed in two layers by 5.0 silk sutures. Sham mice underwent a midline laparotomy without cecum ligation and perforation. All animals received a subcutaneous injection of 0.9% NaCl (1 mL) for fluid loss immediately after surgery.

### 4.3 Sepsis severity and survival study

The sepsis severity following CLP was monitored every 12 h. by recording the clinical scores and survival rate according to a previously established method (Shrum et al., 2014) by two investigators. The murine sepsis score was recorded by evaluating seven individual variables such as appearance (degree of piloerection), level of consciousness, activity, response to auditory stimulus, condition of eyes, respiration rate and quality. A scoring of 0 to 4 was assigned for each parameter depending on the symptoms, higher scores reflect increased severity. Mice were humanely euthanized if the MSS was greater than 21. Mice survival was monitored for 10 days. The mice were divided randomly into Sham, CLP, and CLP+(D+Q) groups. Dasatinib (D; Sigma cat SML2589; 5 mg/Kg) and Quercetin (Q; Sigma cat Q4951; 50 mg/Kg) were dissolved in dimethyl sulfoxide (DMSO). D + Q and DMSO were administered 24 h. before surgery via oral gavage. For molecular studies using plasma and liver separate experiments were performed and mice were euthanized at 24 h. after CLP surgery.

### 4.4 Real‐time quantitative reverse transcription PCR (RT‐qPCR)

Total RNA was extracted from liver using TRIzol reagent according to the manufacturer's instructions (Invitrogen) (Jian et al., 2011). RNA purity and concentration were assessed by absorbance at 260 and 280 nm. One microgram of total RNA was reverse transcribed to cDNA using ImProm‐II reverse transcription system (Promega, USA). Gene‐specific primers were synthesized from Invitrogen (Thermo Fisher Scientific, Inc., CA), (Table S1). Thermocycling conditions: initial denaturation at 95°C for 30 s, followed by 40 cycles 95°C for 30 s and 55°C for 30 s. SYBR Advantage qPCR mix (iTaqSYBR green super mix, BioRad 1,725,122) was used to perform qRT‐PCR (AriaMx, Agilent Technologies). The expression levels of genes were normalized against β‐actin mRNA and expressed as fold change compared to the controls. Relative fold change was determined by computing using the 2−^ΔΔCT^ method.

### 4.5 Biochemical assays/biomarkers of liver damage

Liver function was assessed by measuring plasma alanine aminotransferase (ALT) using commercially available kit as per the manufacturer's instructions (Abcam, MA). Liver Malondialdehyde (MDA) levels were measured using TBARS (TCA Method) assay Kit (Cayman Chemical, MI). Liver MPO activity was measured using an MPO assay kit (Biovision, Milpitas, CA).

### 4.6 Multiplex cytokine array

Blood plasma was analyzed by bead‐based multiplex assay for inflammatory cytokines, chemokines, and growth factors. LEGEND plex mouse inflammatory panel was used according to the manufacturers protocol (Biolegend, 740,446). Samples were acquired on NovocyteQuanteon flow cytometer (Agilent Technologies).

### 4.7 Histopathology—H&E staining

Liver tissues collected 24 h. after CLP surgery were fixed in 10% formalin and paraffin embedded. The paraffin embedded tissues were cut into 10 μm sections, deparaffinized, rehydrated, and stained with H&E using standard protocols. Ten fields per section were captured at a magnification of 200×. The pathological changes in liver tissue were observed under a light microscope.

### 4.8 Senescence‐associated‐β‐gal activity in situ (SA‐β‐gal)

Fresh frozen liver sections (10 μm) were stained with a senescence detection kit (Abcam 65,351), according to the manufacturer's protocol. SA‐β‐gal‐positive areas were assessed and quantified using ImageJ software. On each tissue section, 10–15 regions were quantified at random. Two independent observers conducted the image assessment.

### 4.9 Immunostaining

Fresh frozen liver sections were fixed in 4% paraformaldehyde (Thermo Fisher Scientific, USA) at RT for 15 min and blocked with PBS containing 0.3% Triton X‐100 and 5% normal donkey serum (Abcam, UK, ab7475) at RT for 1 h. Slides were washed thrice in phosphate buffered saline (PBS) and incubated overnight (4°C) with primary antibodies (Table S2). Following washing, sections were incubated with appropriate secondary antibodies for 1 h. at RT followed by further washing with PBS (three times) and mounting with Vectashield mounting medium containing DAPI (Vector Laboratories, CA, USA). Secondary antibodies used were goat anti‐mouse IgG Alexa fluor 488, donkey anti‐sheep IgG Alexa fluor 6471, and donkey anti‐rat IgG alexafluor594. For γ H2AX staining, anti‐phospho‐Histone H2A.X, was used (Table S2). Images were acquired using ECHO Revolve fluorescence microscope.

### 4.10 In situ RNA hybridization—RNAscope

Fresh frozen mouse liver tissue sections (10 μm thickness) were subjected to in situ RNA hybridization using the Advanced Cell Diagnostics RNAscope Multiplex Fluorescent Detection kit v2 (323110, Bio‐techne) as per the manufacturer's instructions. Sections were fixed in fresh 4% paraformaldehyde, for 60 min at 4°C, followed by washing with PBS, dehydration and quenching endogenous peroxidase activity with RNAscope hydrogen peroxide (ACDbio) for 15 min at RT. After washing thrice in PBS, sections were protease‐treated (RNAscope Protease IV, ACDbio) for 30 min at RT and washed a further two times in PBS before incubating with p21 probe for 2 h. at 40°C in a hybridization oven. TSA Vivid fluorophore 570 (Perkin Elmer 323,272) diluted appropriately in RNAscope TSA dilution buffer was used. Nuclei were labeled with RNAscope DAPI (ACDbio) for 30 s and slides were mounted in Prolong Gold Antifade mounting medium (Thermofisher). Sections were imaged on ECHO revolve fluorescent microscope.

### 4.11 Flowcytometry

Liver single‐cell suspension was prepared by enzymatic digestion in DMEM medium containing 5% fetal bovine serum for 30 min at 37°C as per the manufacturers protocol (Miltenyi Liver dissociation kit #130‐105‐807). Debris were removed, followed by red blood cell lysis using ACK lysis buffer (Gibco). Cell suspensions were used for flow cytometry. Senescent cells expressing p21 were assessed by flowcytometry using Alexa fluor 488 anti‐p21 antibody. Other markers used were‐ endothelial cells: Alexa fluor 647 von Willebrand factor antibody; hepatocytes: Coralite555 Asialoglycoprotein receptor; and Kupffer cells: APC anti‐mouse F4‐80 antibody (Table S2). Unstained and single stained cells were used as controls. Samples were analyzed using Attune NxT (ThermoFisher) and analyzed using FlowJov.10.8.1 (Tree Star, USA).

### 4.12 scRNA‐seq library preparation, sequencing, and data analysis

Single cells isolated from 3 to 4 mice (Sham or CLP) were pooled for single‐cell RNA sequencing. Cell suspension obtained from liver was resuspended in PBS (without Ca or Mg) with 0.04% BSA. Cell Viability was assessed using Cellometer Auto2000 (Nexcelom). Samples with viability >80% were loaded in the Chromoium X instrument (10X Genomics) to capture approximately 10 × 10^3^ targeted cells using Chromium Next GEM Single Cell 3′Reagent Kit v3.1 Dual Index form 10X Genomics. scRNA‐seq libraries were generated according to the manufacturer's instructions. QC (quality control) analysis was performed prior to sequencing with the Agilent 2200 TapeStation (Agilent, Santa Clara, USA). Sequencing was performed with Novaseq6000Illumina platform following 10X Genomics guideline.

Sequencing data analysis was performed using Cell Ranger v7.1.0 and the Cell Ranger's version mm10 reference genome (refdata‐gex‐mm10‐2020‐A). The output filtered feature count matrices were further processed and analyzed using R v 4.3.0 and Seurat package v4.9.9 (Hao et al., 2021). Low‐quality cells were removed from each sample if they contained fewer than 500 unique molecular identifiers (UMIs), fewer than 200 features, counts exceeding the sum of the median and 2.5 times the median absolute deviation for either UMIs or features, or more than 15% mitochondrial UMIs. The cell multiplets were removed using the DoubletFinder package v2.0.3 (McGinnis et al., 2019). For normalization and variance stabilization of molecular count data, the Seurat SCTransform method (Hafemeister & Satija, 2019) was employed. Samples from different treatment groups were integrated for clustering using Seurat's canonical correlation analysis (CCA). Clustering was done using the RunPCA, FindNeighbors, and Find Clusters functions with the first 40 principal components and a clustering resolution of 0.3. Nonlinear dimensionality reduction was performed using t‐distributed Stochastic Neighbor Embedding (tSNE) technique with the RunTSNE function implemented in Seurat package. Cluster‐specific markers were identified by applying the FindAllMarkers function in Seurat, utilizing the default Wilcoxon Rank Sum test. Cell types were assigned to clusters using scMRMA v1.0 (Li et al., 2022) with the implemented PanglaoDB reference (Franzén et al., 2019). The normalized counts matrix of MDMs and Kupffer cells was extracted from the Seurat object and filtered to remove genes with zero counts. The MPI and the AMDI were calculated using the MacSpectrum (v1.0.1) online application (Li et al., 2019).

### 4.13 Statistical analysis

Data are presented as mean ± SEM. Statistical analysis was performed using GraphPad Prism 9.0 (GraphPad Software, La Jolla, CA, USA). The percentage of surviving mice and the survival curve were analyzed by Kaplan–Meier curve and the log‐rank (Mantel‐Cox) or Gehan‐Breslow‐Wilcoxon tests using GraphPad Prism. The data was analyzed using two tailed non‐parametric Mann–Whitney test for comparisons between two groups and One‐way ANOVA followed by Tukey test for comparison of multiple groups. *n* indicates the number of mice. *p* value <0.05 was considered statistically significant. Significance (*p* value) is represented as *, where * ≤ 0.05, ** ≤ 0.01, *** ≤ 0.001, and **** ≤ 0.0001 and ns, where *p* > 0.05 for “not significant.”

## AUTHOR CONTRIBUTIONS

**Rupa Lavarti, Lun Cai, Tatiana Alvarez‐Diaz, Thalia Medina‐Rodriguez**: Experiments performed. **Sergei Bombin, Rupa Lavarti, Lun Cai, Raghavan Pillai Raju**: Data analysis. **Rupa Lavarti, Lun Cai, Tatiana Alvarez‐Diaz, Thalia Medina‐Rodriguez., Sergei Bombin, Raghavan Pillai Raju**: Interpreting the data. **Rupa Lavarti, Raghavan Pillai Raju**: Writing the manuscript.

## FUNDING INFORMATION

This work was partially supported by the grants R01AG073338 (RR); R01GM122059 (RR) from the United States National Institutes of Health, I01BX006256 from the US Department of Veterans Administration, and funds from Augusta University. The single cell sequencing was done at the Genomics Core, Augusta University.

## CONFLICT OF INTEREST STATEMENT

The authors have no conflict of interest to declare.

## Supporting information


**Data S1.** Supporting Information.

## Data Availability

The single cell sequencing raw data is available via the GEO accession number: GSE279167.
