# Supplementary material for: Senescence landscape in the liver following sepsis and senolytics as potential therapeutics
Source: Aging Cell. 2024 Oct 23;24(1):e14354. doi: 10.1111/acel.14354 (PMC11709100; doi:10.1111/acel.14354)
Supplement: Supplementary file 1 — Data S1. Supporting Information. [file ACEL-24-e14354-s001.pdf]

Supplementary Tables

Table S1. Sequences of primers used in the study.

**Gene**	**Forward primer sequence (5'-3')**	**Reverse primer sequence (5'-3')**
IL6	GAGCCCACCAGGAACGAAA	AACTGGCTGGAAGTCTCTTGC
IL1β	CCCTGCAGCTGGAGAGTGTGG	TGTGCTCTGCTTGAGAGGTGCT
TNF-*α*	TGC TGG GAA GCC TAA AAG G	CGAATTTTGAGAAGATGATCCTG
$P16^{ink4a}$	TCGTGCGGTATTTGCGGTAT	TAGTCTCGCGTTGCCAGAAG
$p21^{Cip1/Waf1}$	CAGCCACAGGCACCATGTC	ACAGACGACGGCATACTTTGC
P21V1	GGAATTGGAGTCAGGCGCAG	GAACAGGTCGGACATCACCA
P21V2	AATGGAGACAGAGACCCCAGA	GAACAGGTCGGACATCACCA
CDK4	GCTACCACTCGATATGAACCCGTGGCT GAA	GGTGCTTTGTCCAGGTATGTCCGTAGGT CC
cyclin B1	CTATCCTACAGTGAAGACTC	TGCTTAGATGCTGCATAC
cyclin B2	GAGAGTGAAGTCCTGGAA	GTGCTGATCTTCAGGAGT
CXCL1	GCCACACTCAAGAATGGTCG	TGGGGACACCCTTTAGCATC
MMP3	TTTAAGGAAATCAGTTCTGGGCTATA	CGATCTTCTTCACGGTTGCA
CXCL14	GAAGATGGTTATCGTCACCACC	CGTTCCAGGCATTGTACCACT
β-actin	AGTACCCCATTGAACACG	AATGCCAGTGGTACGACC

Table S2. Antibodies used in the study.

Antibodies for Flow cytometry	Host	Dilution	Source	Catalogue
p21 Waf1/Cip1	Rabbit	1:200	Abcam	Ab237264
Asialoglycoprotein receptor	Rabbit	1:400	Proteintech	CL555-11739
Von Willebrand Factor	Rabbit	1:400	Abcam	Ab307377
F4/80	Rat	1:200	Biolegend	123115
**Antibodies for Immunofluorescence**				
p21	Mouse	1:200	Santa Cruz	sc6246
Asialoglycoprotein receptor	Rabbit	1:100	Proteintech	CL555-11739
Von Willebrand Factor	Sheep	1:100	Abcam	AB11713
F4/80	Rabbit	1:200	Proteintech	29414-1-AP

Supplementary Figures

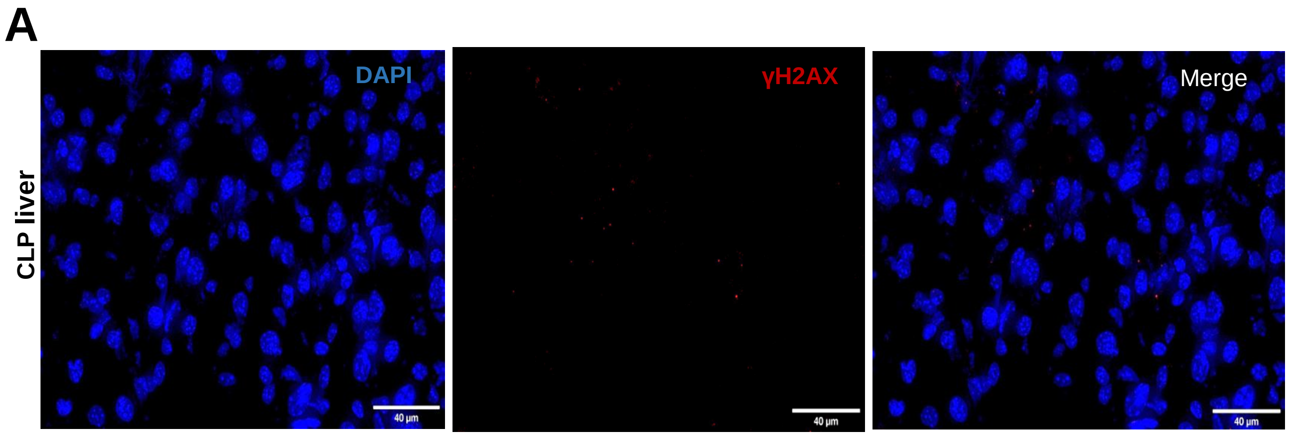

Supplementary Fig-1: (A) Immunofluorescence staining of γ H2AX. CLP liver was negative to γ H2AX foci. DAPI stained nuclei are in blue.

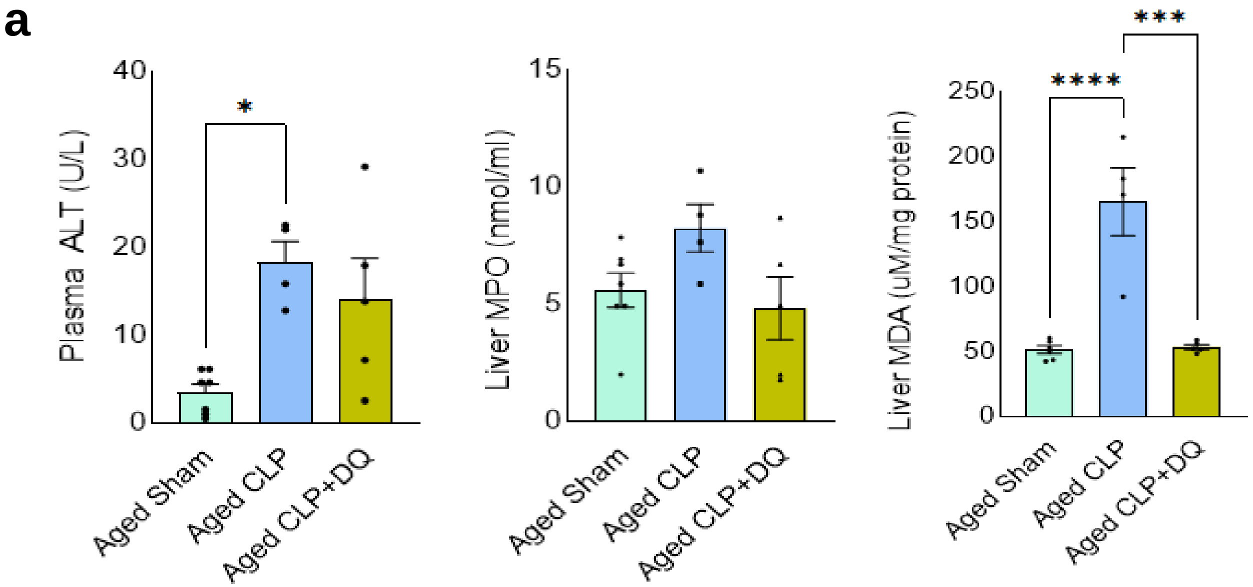

Supplementary Fig-2: (a) Effect of senolytic drugs on sepsis induced liver injury in aged mice. Levels of ALT in plasma, MDA and MPO activity in liver 24 h after surgery. Sham (n=7), CLP (n=4) and CLP mice administered with D+Q (n=5). Statistical analysis: One-way ANOVA with results given as mean ± SEM. ***p<0.001 and **** = p<0.0001.

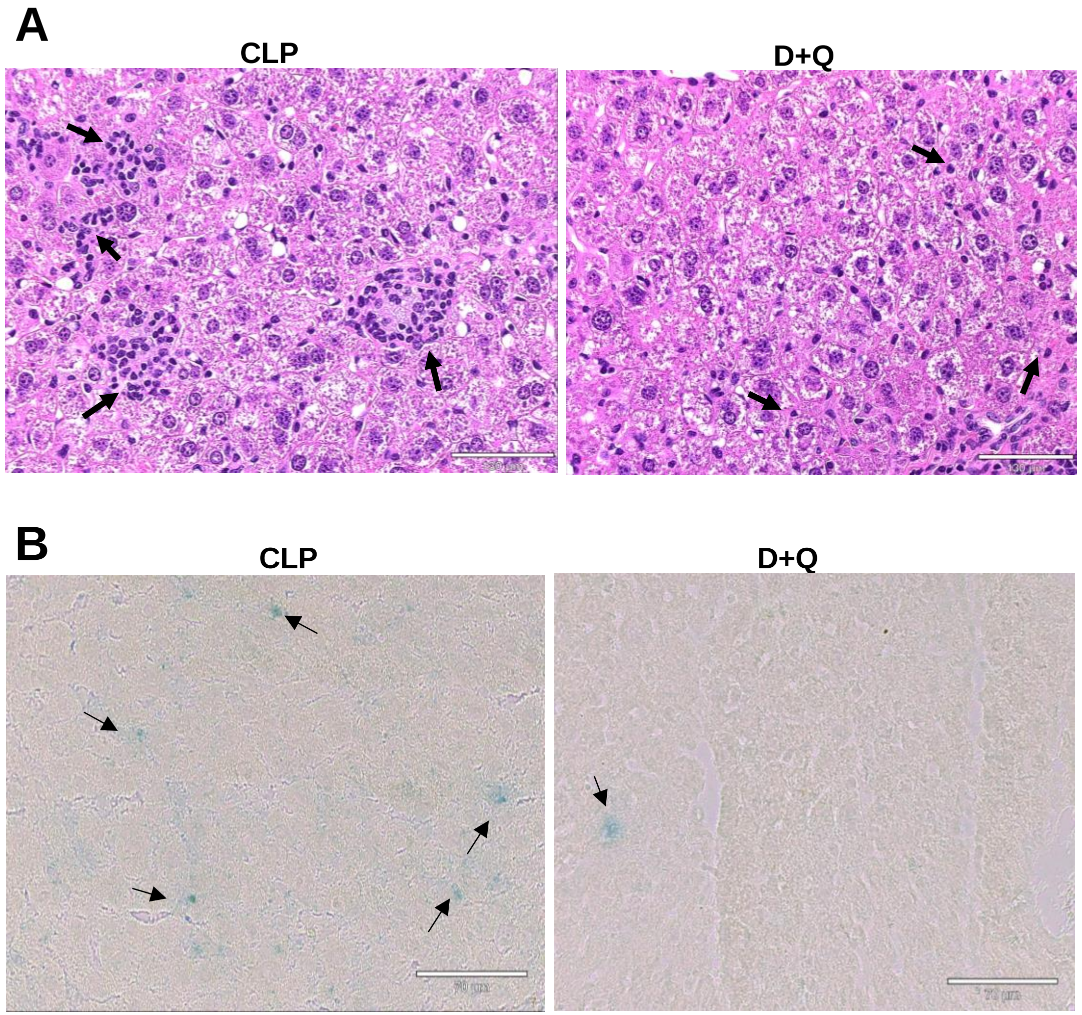

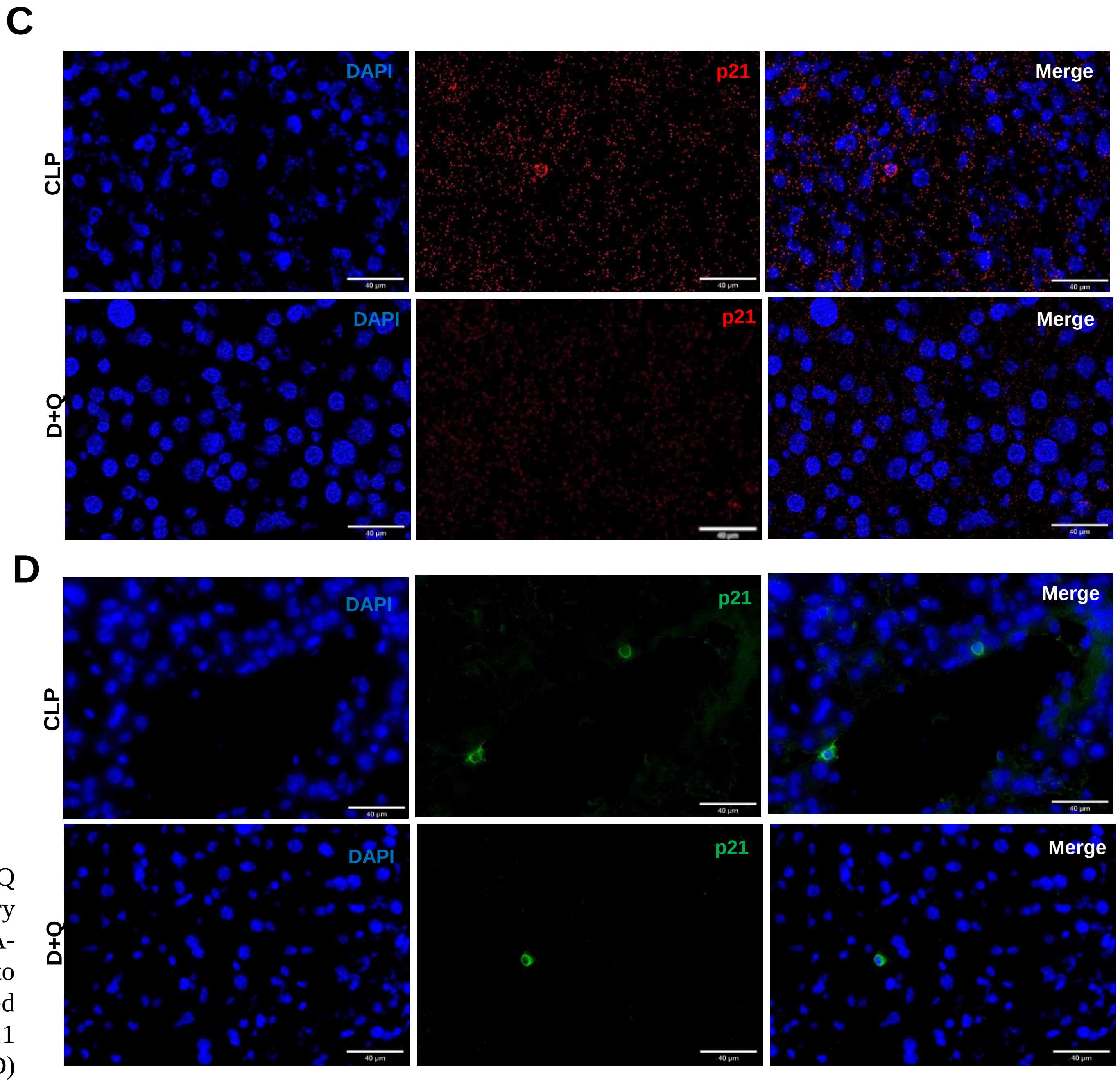

Supplementary Fig-3: (A) Hematoxylin and eosin staining in D+Q liver. D+Q administered mice demonstrated histological recovery with decreased inflammatory cells (black arrows). (**B**) **Senescence associated SA-β–gal staining.** Reduced SA-β–gal intensity (blue) was detected in D+Q administered mice. Scale bar equals to 70 µm. (C) Representative images showing p21 mRNA localization in D+Q treated liver. Nuclei counterstained with DAPI are seen in blue and hybridization with p21 probe is visualized as red punctate dots. Scale bar equals to 40 µm. (D) Immunofluorescence of p21 (green) with DAPI stained nucleus (blue) in D+Q administered mice. Scale bar equals to 40 µm.

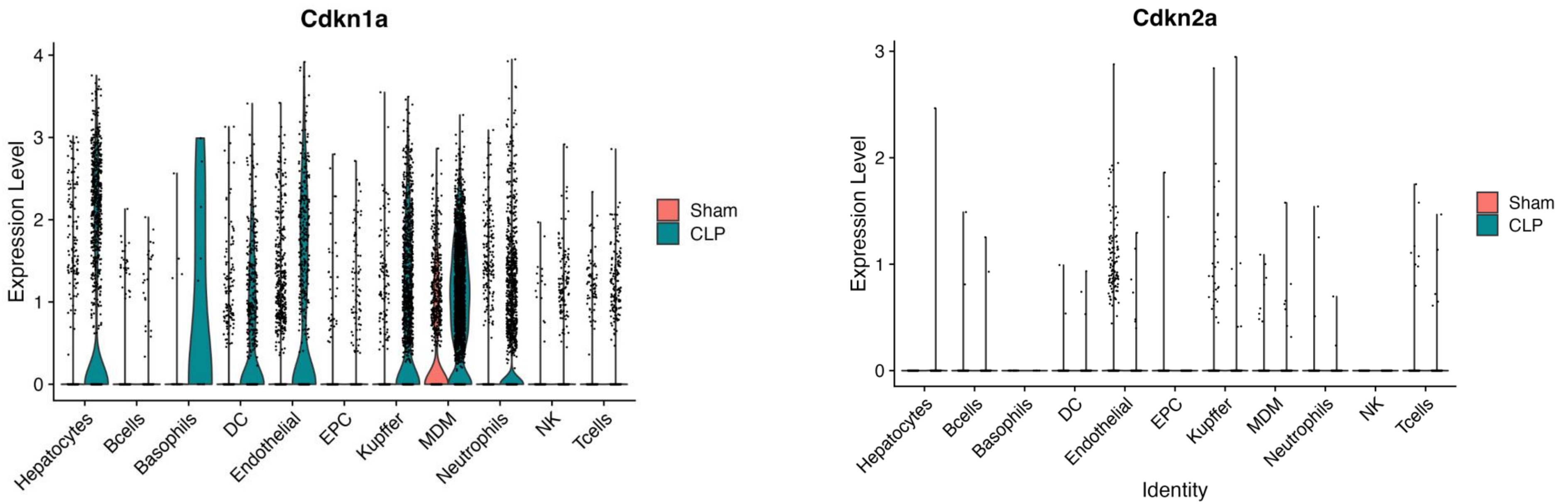

Supplementary Fig-4: Single-cell RNA-seq violin plot showing expression of Cdkn1a (p21) and Cdkn2a (p16) gene signature in different liver cell clusters. In all clusters the expression of p21 is increased in CLP liver as compared with sham liver.

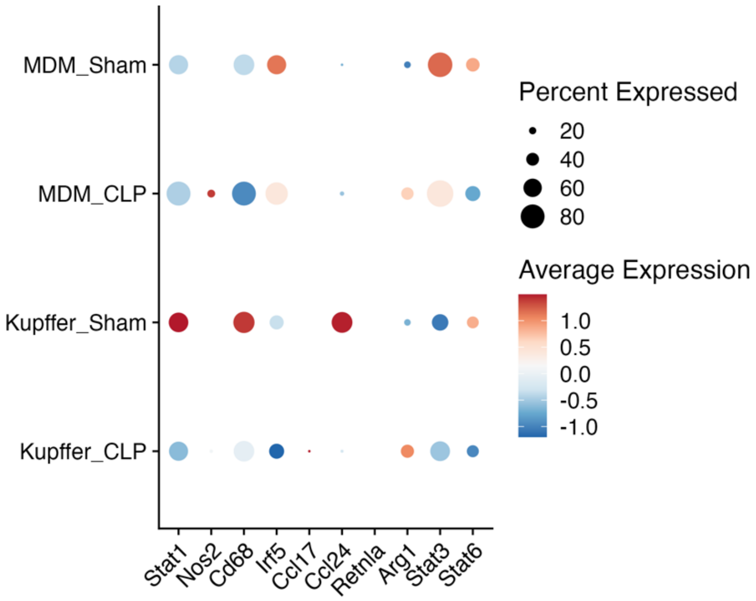

Supplementary Fig-5: Single cell transcriptome profiling of CLP liver. The dot plot representing macrophage subset-specific markers expression in Kupffer cells and monocyte derived macrophages (MDM) of sham and CLP liver.

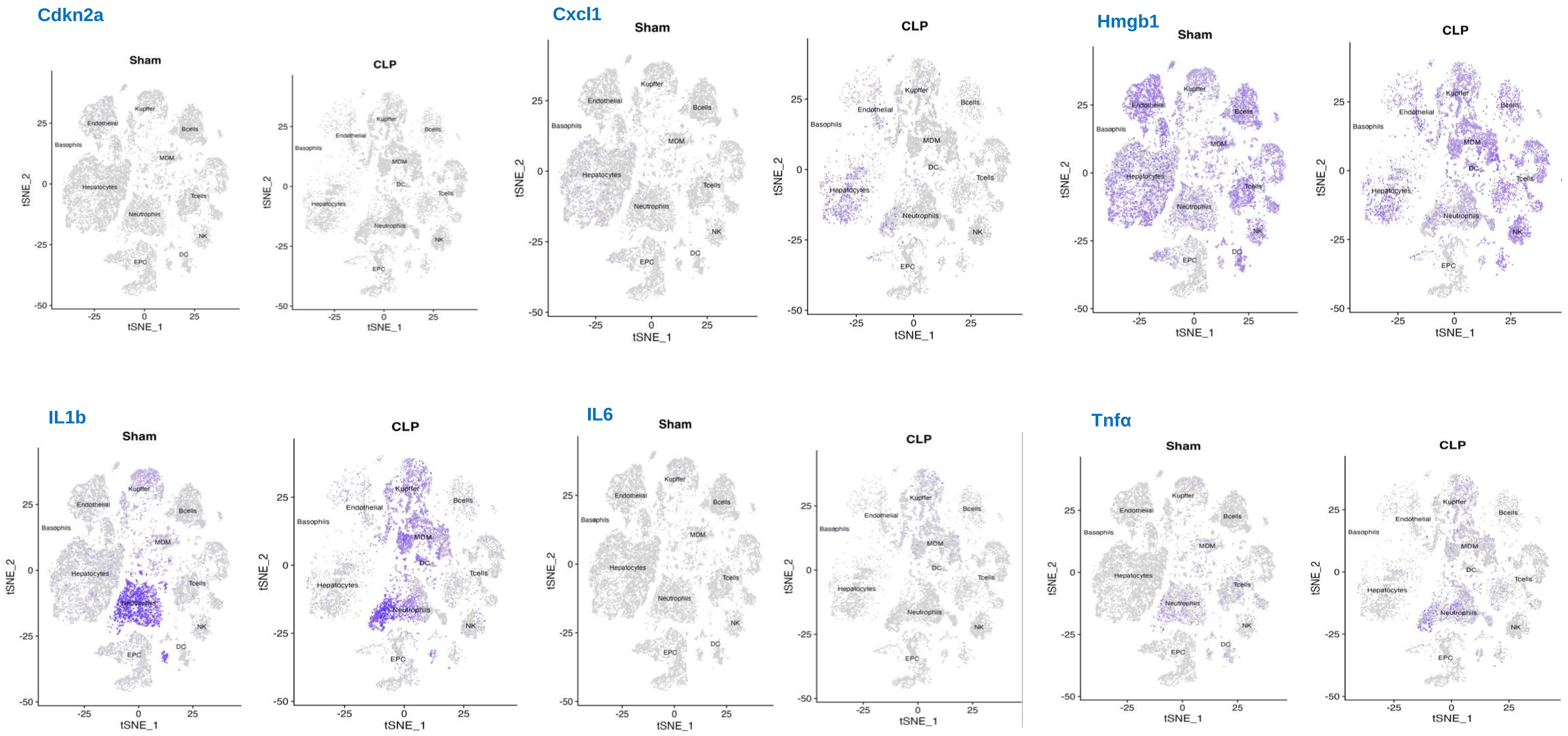

Supplementary Fig-6: Transcriptomics landscape of sepsis liver. (A) *t*-SNE plots showing SASP expression profiles of Cdkn2a, CXCL1, HMGB1, IL-1β, IL-6, and TNF*α*, in sham and CLP mice from the snRNA-seq data.

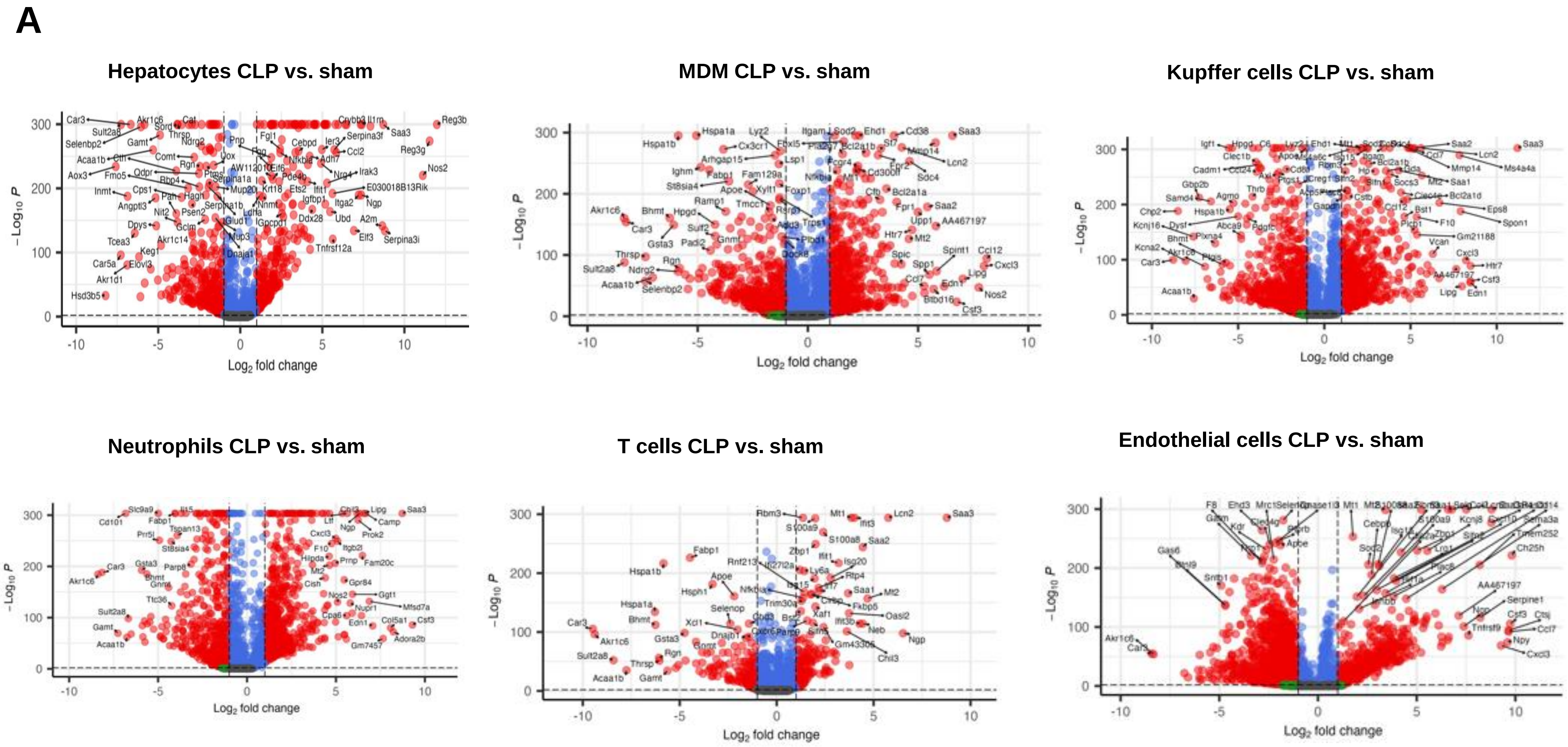
A
Hepatocytes CLP vs. sham
MDM CLP vs. sham
Kupffer cells CLP vs. sham
Neutrophils CLP vs. sham
T cells CLP vs. sham
Endothelial cells CLP vs. sham
$-\log_{10} P$
$\log_2$ fold change

A (continued)

EPC CLP vs. sham

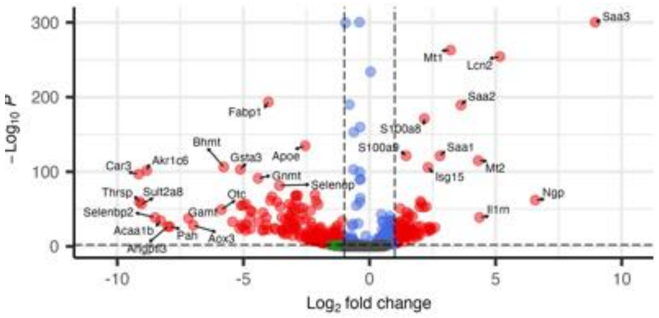

Basophils CLP vs. sham

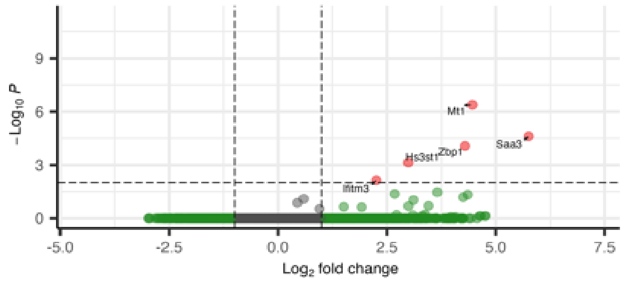

DC CLP vs. sham

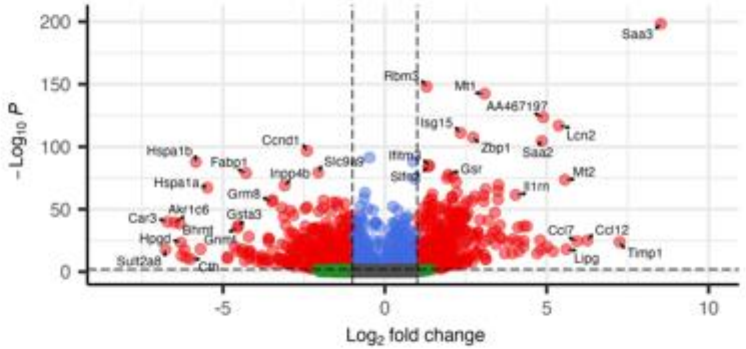

NK cells CLP vs. sham

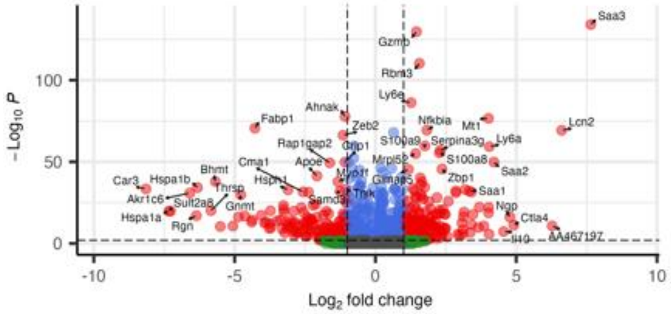

B cells CLP vs. sham

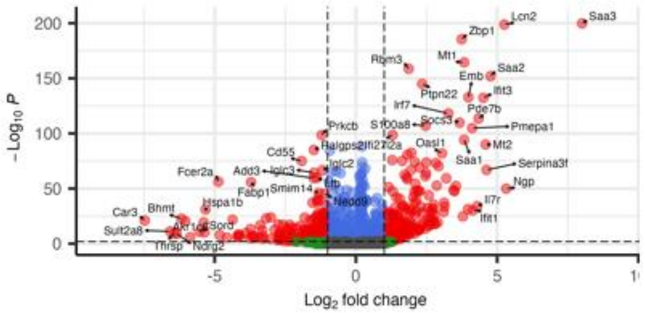

B

Cell Type	Total genes	Up regulated Genes	Down regulated genes
Hepatocytes	2192	337	399
Kupffer	7565	825	1016
MDM	7921	595	515
DC	7713	430	483
Endothelial cells	7200	602	2008
Basophils	5275	5	0
Neutrophils	5198	808	608
T cells	5924	184	177
B cells	5186	237	187
EPC	741	95	182
NK cells	5991	191	163

Supplementary Fig.-7: The volcano plot of differentially expressed genes. (A) Volcano plot showing gene expression changes in response to CLP induction across liver cell types. The X-axis represents the log2-fold change, and the vertical axis represents the P-value differences between CLP and sham. Threshold q-value < 0.01 and Log2FC > 1 for up regulated genes, and q-value < 0.01 and Log2FC < -1 for Down regulated genes. (B) Table represents the differentially expressed genes in liver cell clusters. MDM, monocyte derived macrophages; DCs, dendritic cells; EPC, Erythroid like and erythroid precursor cells; NK, natural killer cells.
